# Monitoring with In Vivo Electrochemical Sensors: Navigating the Complexities of Blood and Tissue Reactivity

**DOI:** 10.3390/s20113149

**Published:** 2020-06-02

**Authors:** Pankaj Vadgama

**Affiliations:** School of Engineering and Materials Science, Queen Mary University of London, Mile End, London E1 4NS, UK; p.vadgama@qmul.ac.uk

**Keywords:** metabolite sensors, sensor biocompatibility, ion selective electrodes, foreign body reaction, O_2_, glucose, lactate

## Abstract

The disruptive action of an acute or critical illness is frequently manifest through rapid biochemical changes that may require continuous monitoring. Within these changes, resides trend information of predictive value, including responsiveness to therapy. In contrast to physical variables, biochemical parameters monitored on a continuous basis are a largely untapped resource because of the lack of clinically usable monitoring systems. This is despite the huge testing repertoire opening up in recent years in relation to discrete biochemical measurements. Electrochemical sensors offer one of the few routes to obtaining continuous readout and, moreover, as implantable devices information referable to specific tissue locations. This review focuses on new biological insights that have been secured through in vivo electrochemical sensors. In addition, the challenges of operating in a reactive, biological, sample matrix are highlighted. Specific attention is given to the choreographed host rejection response, as evidenced in blood and tissue, and how this limits both sensor life time and reliability of operation. Examples will be based around ion, O_2_, glucose, and lactate sensors, because of the fundamental importance of this group to acute health care.

## 1. Introduction

Physiological processes operate under highly dynamic conditions that are controlled by a multitude of biofeedback systems operating on both long and short term timescales. These establish homeostatic control within finite, set, limits. It is the essence of any multi-cell and tissue organism that it is able to maintain relative internal stability in the face of unpredictable, and often undesired, environmental change. Within the cell itself, sensitive surveillance mechanisms recognise status deviation and effect timely responses, on an ultrafast basis if deemed necessary. These responses are all the more effective where delivered through specialist tissues and the major internal organs. Complexities at this cell level are only partially reflected in changes in the extracellular space. However, it is only the changes in the latter that we are able to monitor and assess through available sampling and measurement capabilities. For some extracellular parameters, these changes take place on highly compressed time scales and justify frequent, if not continuous, measurement, for both a better fundamental understanding and the better management of disease. These variables can be considered to be highly labile and their dysregulation representsmajor failure of homeostatic control. Currently, these centre on ions, gases, and small metabolites. Their potential for rapid change is also indicative of the potential value of continuous monitoring to track their trajectory and help titrate therapy. The timing of any therapy is of equal importance to its amount in certain types of critical illness and might influence recovery and survival.

For all practical purposes, only extracellular events are trackable. Moreover, the fractal and multi-organelle architecture of the cell does not readily offer simplified messages that allows for easy conclusions about its status or, indeed, that of the whole individual organism. This remains the case, even if robust microsensors were to become available. A clear differentiation between the normal state and disease is vital clinically and, whatever the biological value of intracellular monitoring, any information thus secured will be a complicated case mix influenced by cell ageing, the cell cycle, the micro-compartment sampled, the reaction of the cell to interrogation, and a myriad of other unknowns that are not easily synthesised into workable diagnostic formulations. The occasional exception is where disease disruption is so overwhelming that it leaves a substantial intracellular signature leading to structural change, such as when an insoluble end product accumulates or a core metabolic process is involved, as in some inherited metabolic diseases.

The earliest efforts with sensors were indeed directed at intracellular monitoring, but for enhancing biological understanding. This involved pioneering work with wire and micropipette electrodes. The latter as ion selective electrodes were particularly prominent, and were used to variously follow intracellular H^+^, K^+^, and Na^+^, e.g., to study cell plasma membrane ion exchanges. A particular interest was in excitable tissues, such as nerve and muscle [1], where ultra-rapid ionic exchanges mediate membrane depolarisation and launch action potentials. Such work initiated our understanding of cell physiology and the cell’s ability to function at high speed. Oxygen was the other intracellular target, enabled through more robust wire-based electrochemical sensors [2]. Priority interest in oxygen emanated from, and still remains, its central importance to the energy economy of the cell and the dire consequences of its deficiency.

The cell, tissue, and whole organism hierarchy gives us a dimensional scale across which we can select monitoring options at the supra-cellular level. For clinical purposes, it is at the whole organism end of the range that we can see direct clinical value. This larger scale is fortunately readily accessible to us via the blood circulation, accommodating invasive probes. Whole body physiology targeted this vascular space in order to understand change in the intact organism. The current medicine paradigm remains the use of blood as the ultimate pre-mixed representative of whole-body change. Whilst blood is core to our disease understanding, it is still an approximate messenger, being differentially affected by various tissues and with a compositional change that may also only be a diluted version of events at local level. Our access and assay technologies in the clinical laboratory are still inevitably directed at blood. A typical diagnostic biochemistry repertoire is 200 parameters, a triumph for analytical science [3], but one less attentive to following rapid change other than by more frequent discrete measurement. Yet, we already know there is advantage to continuously monitoring oxygen, certain ions, and metabolites in blood.

Other fluids, such as urine and CSF, might have discrete measurement value, but continuous monitoring is unlikely to be of added benefit. The tissue biopsy for targeted analysis lies at the opposite extreme end of the scale for repeat measurement need [4]. Our developing blood biochemistry repertoire has made it possible to gain a better picture of the timescale for variation. Thus, homeostatic control, far from creating a biochemically static environment, effects a pattern of pre-set cycles and modulations. Chronobiologists have elucidated such patterns for the healthy state, segregated variously into ultradian, circadian, and also longer-term and less well understood forms of biorhythm [5]. In disease, there is not only a deviation from normal set points for a biochemical parameter, but moment to moment fluctuations that might change in dynamic character and could contain added information. This has gone unrecognised because continuous tracking is not available, and the minor fluctuations nominally trivial. Rapid change possibilities were understood in relation to monitored blood gasses: pH, pO_2_, and pCO_2_ [6,7]. However, from what we now know of glucose and lactate, these and other intermediate metabolites may well also exhibit rapid fluctuations.

**Figure 1:**
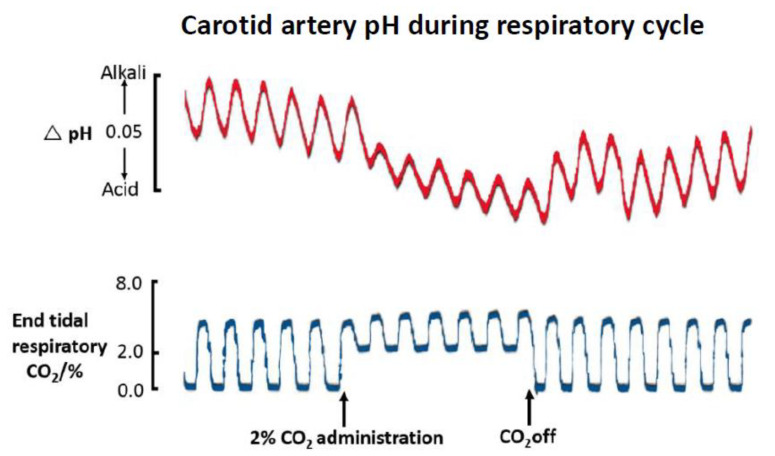
Arterial blood pH monitored extracorporeally at a carotid artery loop in an anaesthetised cat using a glass pH electrode. End tidal CO_2_ was monitored by an infrared CO_2_ analyser. Administration of 2% CO_2_ led to increased end tidal CO_2_ and a drop in arterial pH. The pH trace also shows breath to breath arterial pH oscillations. Adapted from [12].

In early examples relating to ions, continuous monitoring of blood was undertaken in animals using ion selective electrodes (ISEs) in early physiology studies [8,9,10,11]. Striking speeds of change were seen. These studies were remarkable in being well before the advent of the microfabrication toolkit for reproducible sensor miniaturisation. In one example, rapid change to arterial blood pH was shown as a result of increased inspired CO_2_ composition (Figure 1) [12]. The conversion of CO_2_ to carbonic acid by carbonic anhydrase in red cells, we know, is rapid, but its manifestation as a matching fast outcome seen through blood [H^+^] is a useful dynamic indicator. More remarkable, perhaps, was the observation, as in this study, of breath to breath pH oscillations due simply to normal breath to breath pulmonary tidal pCO_2_ variation. The oscillations are stable, and remarkable for being observable in the highly buffered entity that is whole blood. Interestingly, the amplitude of these oscillations parallels respiratory CO_2_ excursions. The nature of these oscillations in respiratory illness and compromised blood buffering remains an unknown, but could provide new clinically relevant information. Only a fast response ISE with its millisecond response has the capability to unmask these hidden variables. Sensor advances, combined with signal processing, might provide a valuable step forward in extracting and processing such hidden information. Technically, the active membrane components used in ISEs have changed little, a tribute to the early chemists. However, other newer design iterations could be usefully pursued. An ion selective membrane for bicarbonate ion has yet to be made, yet bicarbonate ion has huge importance for the acid-base status of the body in disease, and its dynamic variation is unknown.

A classical basis for the in vivo sensor is its ability to follow a trend and thereby to pick up deviation early, even whilst a parameter remains within the bounds of normality. With wider deployment of such in vivo sensors, there will be greater identification of early variation and of the hidden dynamic patterns that are linked to disease. Minor oscillatory and other minimal changes will need high resolution sensing. Rapid response will also be crucial, as shown with pH. However, on the speed of response, it is probable that a slower biosensor tracking of metabolic fluctuations will still be sufficient. Oscillatory cycles for basal insulin and glucagon have been observed in blood, and are relatively slow, on timescales of minutes. They also have a synchrony with a glucose oscillation of similar periodicity [13], though the glucose cycling has an amplitude of merely 0.05 mM. Future sensors that are able to track such minor cycling behaviour could allow linkage to be made to metabolite control and its dysregulation.

The dynamics of oxygen change at tissue level were the focus of some early studies. These helped to unravel interrelationships between oxygen delivery centrally and localised tissue oxygen uptake. The latter is modulated by the local microcirculation, itself in a state of dynamic variation. Silver [14,15] investigated normal and solid tumour tissue pO_2_ using microelectrodes; Lubbers [16,17] used multi-wire electrodes and tracked both oxygenation variability and microenvironment heterogeneity. The time dependent shunting of O_2_ was regularly seen, involving complex cycling topologically connected to the organisation of the vascular bed. The interstitial tissue space was thus demonstrated, for oxygen at least, as the seat of complex gradients, which alsoreflected the balance of cell respiration against tissue level fluctiations in blood supply.

A physiology study may only require a sensor to operate for a limited period, and this also under highly supervised conditions. The progression to medical use poses a more severe challenge where simplified, robust operation is the key. Technical refinements and miniaturisation have moved the field along in analytical chemistry terms, but stable in vivo deployment and local tissue communication has proved to be a more protracted challenge. In the absence of reference methods, monitored output is always a combination of true metabolic readout and an uncertain, artefactual change due to local implant site tissue change, including that leading to fouling on the device sensing surface. The balance might well be towards a meaningful readout and not to artefact, but the latter can prove a remarkable mimic of real change. The communication interface with tissue or blood is thus a weak link. Interface stability is under constant threat from assembled biological reactivity that changes the very environment intended as the sampling window into whole body changes. Our understanding of the complexity of both the reactive process and it structural outcomes is still limited. Innumerable sensor design approaches, including of new transduction routes, have been reported to combat the adverse effects found in the host environment, but success has been partial, at best. For tissue, especially, the sensor is merely another foreign body intrusion, and it is of no consequential difference to the host biology as to whether it has been architectured as an ‘intelligent’ material or some other standard, unreactive biomaterial. Emphasis is needed now on the biology to find a route to more reliable monitoring and the promise that it holds. Recent work has begun to consider these extra-sensor processes and their key elements. The balance of work, though, still does not reflect the centrality of the biological question. Existing biomaterials research certainly provides a guide, but sensing research needs its own approach now. On this aspect, the review summarises the response basics of blood and tissue, and how these can affect sensor performance. Examples of dynamic monitoring, further, show why the effort is worthwhile. Therefore, the review is really about the bio-interface. Detailed descriptions of transduction chemistries and electrode designs can be found in the many reviews already published on these aspects.

## 2. Sensors for Continuous Intravascular Monitoring

### 2.1. Ion Selective Electrodes

The ISE has two intrinsic advantages for monitoring. Firstly, it responds on the basis of surface ion binding and does not require slow diffusive access to deeper structures. This delivers a response within milliseconds. Secondly, response is on the basis of equilibrium ion binding, so a maintained ion flux to the device is not needed. The first allows for unmasking of rapid transients (Figure 1), the second is much less affected by surface fouling, other than possibly by a slower dynamic readout or if a deposited layer itself has charge properties that alter ISE potential. The special feature to recognise in practical monitoring is that ISE emf response is log-linear and, for a monovalent cation M^+^, this is approximated in the Nernst equation by:

(1) $$E=E{}^{\circ}-2.303\frac{RT}{nF}Log\left[M^{+}\right]$$

where *R*, *T*, *F*, and *n* have their usual meaning. This means that concentration resolution that is based on the emf will be considerably better at lower concentrations than at high ion levels. Only because of this can we differentiate pH 7.35–7.45, the reference range for blood pH. The resolution of millimolar concentration ions is also readily achievable, but measurement is more challenging for a divalent cation, such as Ca^2+^. Not only is the Nernstian slope of 61mV/decade (body temperature) halved, but finer emf resolution is demanded than for Na^+^/K^+^; the reference range for blood Ca^2+^ is a mere 1.05–1.3 mM. A further practical issue for blood use, likely to be made more complicated in a tissue matrix, is changes to the liquid–liquid junction potential at the reference electrode. This is a combined function of sample ionic strength/colloid composition through to cellul sedimentation and streaming potential, viz an encounter with flowing blood. Finally, it should be born in mind that the ISE responds to ion activity and not concentration; to that extent, it is a true thermodynamic measurement. The activity coefficients at biological fluid ionic strength are around 0.65, so not only are matched calibrants critically important to measurement, but any background ion change will affect the measured values simply via activity change, even if true ion concentration is unmoved. Despite these uncertainties, ISEs have seen effective clinical laboratory use through use of meticulous quality control and use of reference samples. Further extrapolation to in vivo monitoring makes this challenging, especially without sample dilution, so it is fortuitous that clinical value here is not conformity to accuracy *per se* as needed for standard clinical decision making, but in picking up trends fast and in their timely management.

ISE biological sample use has been mostly without modification to device membranes. Indeed, a material as unpromising and as bioincompatible as a glass pH membrane is usable in biological fluids in the first place because of its independence from continued ion flux for a stable reading. The more relevant issue for ISEs in vivo, though, is the potential toxicity of incorporated ionophores and plasticisers. Though quantitatively small in amount, they are biologically active and toxicologically risky; the standard plasticiser 2-nitrophenyl octyl ether, for example, is pro-inflammatory and active ionophores will have cell membrane effects.

In the main, in vivo studies have focused on K^+^. It is of predominantly cellular origin, and its abnormal release can both reflect and cause instability in excitable neuromuscular tissues. In an early animal study, Hill et al. [18] devised a flexible intravascular catheter sensor for K^+^ with a membrane comprising the K^+^ ionophore valinomycin in PVC and achieved low drift monitoring with a catheter mechanical compliance suited to intravascular use. A tip diameter of 0.6mm enabled safe small vessel insertion without flow obstruction. Femoral vein catheterisation in humans was achieved with low drift (<3 mV/h) and enabled monitoring of rapid K^+^ efflux from muscle during exercise; even K^+^ transients of <0.1 mmol/L were picked up [19]. A high pressure vessel poses an added practical catheterisation risk, but arterial catheterisation has also been reported. In an animal study using a carotid artery catheter, rapid K^+^ release from carotid body oxygen chemoreceptors was monitored during their hypoxic stimulation [20]. The benefits of precise localisation of a sensor tip were shown using a catheter advanced into the coronary sinus of the heat in human studies. This blood vessel serves as the common venous conduit for blood draining the heart. In patients suffering from myocardial ischaemia, K^+^ transients were shown that would have been undetectable within the general circulation [21]. Moreover, K^+^ release from myocardium correlated with the severity of ischemia [22].

Rapid blood Ca^2+^ transients have also been shown using indwelling catheters. In one report, a synthetic Ca^2+^ ionophore was used with a plasticised PVC membrane [8]. In a study on dogs, cardiac venous drainage monitoring showed Ca^2+^ perturbations due to injection of an ionised X-Ray contrast agent [23]. With glass being unacceptable for in vivo use, pH catheters have been made using H^+^ affinity polymersand ionophores, e.g., octadecyl isonicotinate [24]. When applied to coronary sinus monitoring, blood pH change could be tracked in cardiac ischaemia patients [25].

Much less has been accomplished with the fibreoptic monitoring of ions in medicine. Few chromionophores are available for high selectivity binding to alkali cations, where ion binding is both selective, and leads to high resolution optical change. Additionally, there is the risk of reagent leaching and photobleaching, more likely during extended operation. However, fluorescent weak acid/base dyes are readily available, and some have allowed monitoring. This includes of tissue [26] and intravascular pH monitoring [27] in animal studies. In the latter, immobilisation of the fluorescent dye within a sol gel matrix provided protection from photobleaching and use of a haemocompatible outer 2-Methacryloyloxyethyl phosphorylcholine (MPC)-cellulose membrane offered protection from blood clotting, observed overeight hours. The operational advantage of an optical route is indifference to background electrical noise and avoidance of a reference electrode. Ratiometric measurement of fluorophore fluorescence with pH offers internal self-referencing to compensate for extraneous artefactual optical changes. Miniaturisation is also readily possible without compromise to fibre robustness, flexibility or integrity. Because response depends on equilibrium binding, biofouling, as with ISEs, should be less of a problem, but response times are outer membrane diffusion constrained and, hence, much longer than for ISEs.

### 2.2. ISE Biocompatibility

The active ionophore of an ISE is typically a high affinity, high binding reversibility agent, and if able to exit the membrane could pose a risk in vivo. The direct consequence would be permeabilization of the cell plasma membrane. The quantities used for small in vivo ISE membranes are clearly insufficient for systemic toxicity, but they could still pose local tissue risk; regulatory approval in any case would necessitate extensive testing compliant with standards. Discovery research for new ISE membranes will be able to extend our analytical repertoire for ions, but should preferably now combine biological with chemical screening. Cánovas et al. [28] undertook such combined evaluation studies of ISE membrane components and tested cytotoxicity in vitro, notably for valinomycin, the most efficient K^+^ ISE ionophore to date. Given its potential toxicity, the antibiotic mutacin, a polycyclic peptide, was suggested as a possible alternative ionophore for K^+^. Much, of course, depends on of the extent to which an ionophore will leach out, and this, in turn, will be a function of membrane permeabilization via the co-entrapped plasticiser. The plasticisers assessed in this study showed varying degrees of toxicity, and reinforce the desirability of pre-use screening. The polymeric ISE membrane itself should not be a toxicity concern. A polymer is only really toxic in so far as it releases its small molecule constituents. This could possibly arise from polymer biological degradation, as seen with polymeric biomaterials. Indeed the reactive implant site has high degradation potential with its constituent cellular hydrolytic enzymes, lowered pH, and free radical release from activated phagocytic cells. So again, biological screening needs to be part of any new polymer development, other than, possibly, in the case of established PVC or polyurethane.

ISE surface modification and coating for safe retention of diffusible membrane components is unlikely in future, given the parallel need for target ion access. However, a possibility does exist for reducing surface biofouling by a coating. Pharmacologically active agents for stabilising blood platelets at the surface could also help to mitigate fouling, and surface hydrophilic layers, such as of polydopamine [29], and hydrogels, such as poly(2-hydroxyethyl methacrylate) (pHEMA) [30], have been reported. Zwitterionic phosphorylcholine is an integral component of the outer red cell membrane surface, and when used, can confer a high degree of haemocompatibility [31]. Other biologically inspired molecules have also shown effectiveness. Surface immobilised heparin [32] has been used, and a NO adduct in a membrane released thrombus countering free NO [33]. Heparin works through binding antithrombin and thereby concentrating its anticoagulation effect if used at a surface, and the ubiquitous signalling molecule NO provides surface protection through its platelet passivating action. As with any small molecule agent, including mediators used for second generation enzyme biosensors, whenever a new component is contemplated, the risks of agent toxicity also need to be considered; the analytical benefits alone do not confer in vivo usability.

A further factor for any intravascular component is progressive thrombosis at a point other than the sensing surface. The result could be flow blockage or disseminated thrombi to a distant tissue location. Materials for surface biocompatibility are often only tested over limited periods, and usually in vitro, so, whatever promise is shown might not transfer to in vivo deployment. One complicating factor is physical vascular flow; platelets are highly environmentally sensitive, and they become activated even by local flow turbulence, so surface deposits may occur due to smooth flow disruption, regardless of any high material surface haemocompatibility.

Future designs for ISEs, if used intravascularly, will need an integrated approach, whereby not just the sensing surface, but also the flow compatibility of the entire construct in a confined blood vessel will need optimisation. The starting point, though, is a recognition of the scale of this in vivo biological challenge with blood. Testing with blood sub-components in focused studies and under controlled conditions can only serve for initial understanding. There is a limit to the practical value of such a reductionist strategy, with a resultant ever-present risk of overoptimistic commentary about some new material or surface delivering almost completely what is needed. Whole blood is a high alert, rapid response system that features multiple cooperative systems. It is able to harness a combination of cellular effectors just like a tissue, andso is not dependent simply on diffusible humoral signalling agents. It has evolved to recognise, package, and potentially degrade any foreign surface intrusion, both as part of a fail-safe haemostasis and as a means of partitioning any 3D object exposed within the circulation. Later parts of the blood response, in fact, begin to resemble those of tissue more closely. From the first, transient, foreign surface encounter, it generates a coagulation cascade for thrombus formation which accelerates through multiple enzymes and finally reaching an explosive rate in the mass generation of the final fibrin crosslink layer. It also utilises a parallel complement cascade that delivers surface coating protein (C3b) that promotes phagocytosis, amongst other effects, and the promotion of inflammatory change in the clot. An ISE in vitro to in vivo transition is thus challenging and warrants greater balance in blood vs. sensor basic research if the early gains of sensor design are to be translated to routine clinical use. It might seem attractive to consider an intermediate solution with the use of an extracorporeal sensor as part of a controllable external blood circuit. However, this also is not a simple solution; whilst greater control over blood flow dynamics, coagulation, and calibration are achieved, complicated pump flow control and sterility protection are now needed. A cumbersome platform and secure fluidic supply can allowusage only be appropriate in high dependency clinical care environments.

### 2.3. Oxygen Electrodes

After the adaptation of O_2_ polarography to in vitro blood use, via the Clark electrode, the measurement principle has changed little. Electrodes use gas selective membranes that are able to reject other solutes and ions while retaining an electrolyte film for stable cathodic O_2_ reduction. Also excluded are cells and colloids from the sample so eliminate working electrode fouling. The electrochemical reaction is substantially more complex than the summarising four electron, −0.65 V (vs. Ag/AgCl), reaction typically cited:

O_2_ + 4 e^−^ + 4 H^+^ → 2 H_2_O (2)

It is dependent variously on oxygen adsorption, surface reactions and electrode material catalytic properties, and is affected by solution conditions—alkaline vs. acid. Gold is the preferred working electrode for oxygen. The electroreduction process here involves first the adsorption of hydrated O_2_, then converted to hydroxyperoxide (OOH), a key intermediatesurface [34]. Subsequent reduction may go by two 2e steps with H_2_O_2_ intermediary or by a combination of these with the above 4e reaction. These also only represent some of the possible electron transfer reactions. The practical aspect of H^+^ utilization is a possible pH induced drift in response due to alkalinisation of the low volume electrolyte film of the Clark electrode. The electrode adsorption-reaction cascade here is also a reminder of the ever-present risk of surface contamination effects. Low molecular weight species in biofluids, especially, have the capacity to adsorb and disturb the catalytic surface, so such adsorption is not limited to just macromolecules. This is also what Leland Clark effectively avoided with his gas permeable outer membrane. This is less easy to avoid with porous membranes and a problem therefore exists for the glucose sensor (*vide infra*). Bbiocompatibility problems are the consequences of diffusible species warrant study, especially since only in the Clark electrode with its blocking polypropylene or PTFE can internal contamination be totally discounted.

Oxygen sensor miniaturisation for intra-arterial use has been achieved [35], but it is difficult given the need for seamless attachment of a relatively inert hydrophobic membrane barrier material to the body of the device. Equally, surface chemical modification forhaemocompatibility is difficult; functionalisation here needs harsh treatment. Also, any deposited coating might delaminate, andany residual exposed hydrophobic surface, will promote fouling through the extra tendency of hydrophobic surfaces to denature adsorbed protein. Denaturation is more likely to trigger a greater host response than with a non-denatured protein. Surface fouling for an oxygen sensor is important because its response requires a continuous, stable flux of oxygen for a plateau response, in contrast to the ISE.

**Figure 2:**
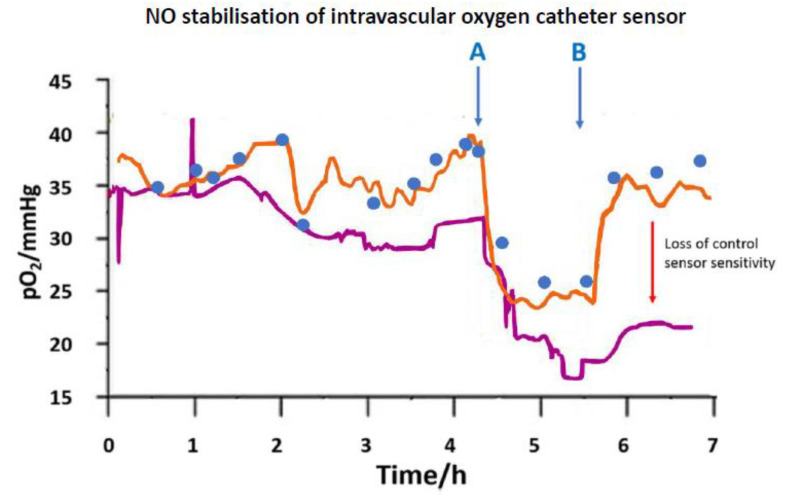
Double lumen intravascularly placed O_2_ catheter with haemo-protective NO delivery used in rabbit jugular vein. (**-**) NO flow protected O_2_ sensor, (**-**) control O_2_ sensor. Blue filled circles are intermittently sampled venous blood pO_2_ values assayed by in vitro analyser. (**A**) 100% inspired O_2_ was switched to 21%. (**B**) Return to 100% inspired O_2_ Adapted from [38].

Vascular catheters as a monitoring route have been reported [36,37] in early studies, and a more structurally refined catheter model has used the catheter wall itself, e.g., silicone, as the gas membrane. NO release at such an electrode offered partial suppression of surface coagulation. In one example, a double lumen silicone cannula was used where NO was electrochemically released from the second lumen containing a nitrite reservoir [38]. Here, stabilised O_2_ output was seen during acute monitoring of hypoxia (Figure 2). Surface coagulation, even though not entirely eliminated, could still be made sufficiently low for extended monitoring subject to a design for sustained NO release. At such a blood contacting device there is also the risk, in principle, that blood cells, notably nucleated cells, highly active metabolically, can act as an oxygen sink, depressing measured O_2_ values. Platelets are also active in this regard, though not the mitochondria deficient RBCs. Ultimately, the blood interfacing problem could be amenable to resolution through synergistic use of locally delivered and surface immobilized anticoagulant agent, along with refined catheter shape to sustain normal blood flow profile. The extracorporeal answer to this is a multiparameter system available for neonatal use (VIA LVM Blood Gas and Chemistry Monitoring System, VIA Medical) [39]; reliability here is achieved through blood flow alternating with heparinised calibrant solution.

Numerous dye functionalised fibreoptic sensors for intravascular pO_2_ monitoring have been reported and for a period available in a commercial clinical intravascular catheter [37], where a triplet of pH, pO_2_, and CO_2_ was monitored. Oxygen monitoring is universally based on dye fluorescence quenching. Oxygen reversible binding to the dye leads to non-radiative transfer of energy and thus reduced fluorescent emission/lifetime (Stern–Volmer relationship). This approach has a huge theoretical advantage over an electrochemical sensor in that a sustained flux of O_2_ is not needed for response and so external transport constraints in vivo are reduced. Nevertheless, problems of surface coagulation in blood and the dangers of thrombus generation are not avoided, and these can stilllead to artefactual output change in measured pO_2_. Measurement uncertainty is further compounded by the catheter wall effect, where catheter tip impaction against a vessel wall blocks off sensor surface blood contact. Otherwise, practical performance is similar to that of electrochemical sensors, with dynamic response, for example, being set by the membrane barrier interposed not the internal chemistry. The similarity of reliability problems in blood for the contrasting type of devices attests to the often limited benefit achieved with radical changes in transduction method.

## 3. Blood as a Reactive Sample Matrix

### 3.1. Protein Surface Interactions

**Figure 3:**
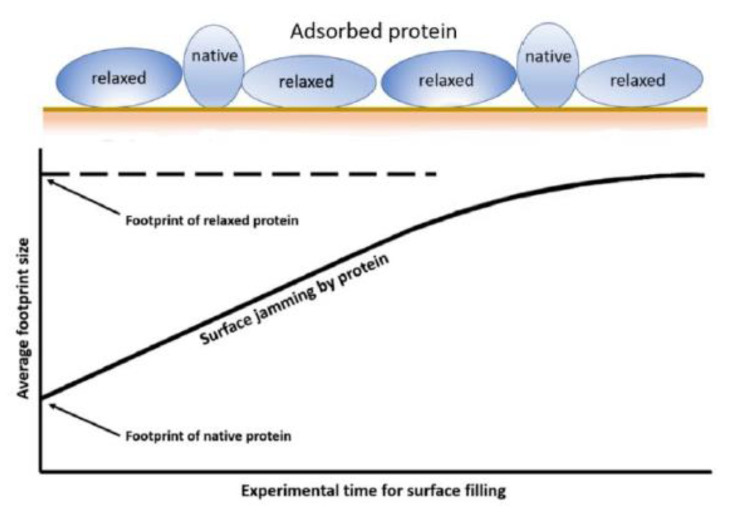
Schematic of progressive relaxation of adsorbed protein layer and increase in surface occupancy per molecule over time. Amount of protein needed for total confluent coverage (jamming) of surface is reduced as time of experimental observation increases. Adapted from [40].

Blood-surface recognition utilises multiple, complex pathways that are, as of yet, incompletely understood. Both plasma proteins and the formed elements of blood, other than RBCs, have a high tendency to adsorb to surfaces. Protein deposition commences within milliseconds, and is later amplified through complement and coagulation cascades that deliver high mass surface coatings. This is the start of the thrombus and, though it might be structurally indeterminate, it advances through highly organised, controlled pathways. The speed and multi-factorial nature of the process makesexperimental study difficult. Consideration of the idealised situation of a single protein as a monolayer provides a model to understand the initial events. Immediately after the deposition and attachment of a protein to any surface, conformational remodeling is initiated due to non-covalent binding interactions with the surface and desolvation changes. Essentially, this is protein denaturation, which leads to peptde chain unravelling and molecular dimensional expansion. Thermodynamicallly, enthalpy lowering drives these surface attachments, but, since attachment also leads to a reduced entropy, in order to compensate, available free loops unravel to thereby increase entropic freedom. Such a molecule has been considered to have a ‘loopy’ conformation. It’s the result is that its surface footprint increases in area. The extent of this unravelling process depends on time and, so for a given mass, the area occupied will increase (Figure 3) [40]. If in a biofouling study, the assessment period is a short one, then in this idealised situation, there will be a higher surface mass per unit area than if the experimental time scale is a long one when the protein molecules have expanded with fewer needed for full coverage, ie jamming. This makes for uncertainty in study comparisons. In the limit, all of the molecules unravel and molecular surface density reaches a finite minimum.

The adsorptive behaviour of proteins from blood is orders of magnitude more complicated, but still driven by the thermodynamics. This complexity is partly summarised by the Vroman effect [41]. This, in any multi-protein system, there is competitive protein surface binding and exchange, not yet fully understood. Early protein adsorbates from high concentration proteins are later displaced in this model by slower arrivals with stronger surface affinity. Typically, here, fibrinogen eventually replaces albumin. This shifting protein interface creates a changing contact surface, and is also the trigger for later biologically mediated cell and fibrin coagulum deposits. Protein denaturation is inevitable at a surface, and this is also a stimulus for blood activation through its presentation of new protein motifs (epitopes). Later, a different type of protein depositionbecomes activated, via the complement cascade; opsonization, andthis is designed to facilitate phagocytosis by polymorphonuclear leucocytes.

Efforts to ultimately achieve zero protein adsorption would seem unrealistic with respect to effectiveness against intact blood biology. Brash suggested an interesting alternative [42]. This envisages that if deposition cannot be avoided, then surface directed selective protein deposition might prove effective. Thus, a surface might be able to selectively invest itself with a defined functional property, such as fibrinolysis (plasminogen adsorption) or anticoagulation (antithrombin adsorption). Reports on haemocompatible sensor surfaces still indicate a continued quest for the single ‘magic bullet’ solution where none may exist. Nevertheless, general rules may be derived from such studies, such as general rules for hydrophobic/hydrophilic balance for lowered fouling and specific surface chemical motifs that link to complement activation [43]. The surface protein profile together with its later remodeled form [44] presents the real final contact layer for all the subsequent cellular processes organised by blood. So from the start, the original engineered or chemically designed surface ceases to be the direct material. Despite this masking, blood recognition continues and its reactivity remains as long as the device is in place. 

### 3.2. Blood Biological Reactivity

**Figure 4:**
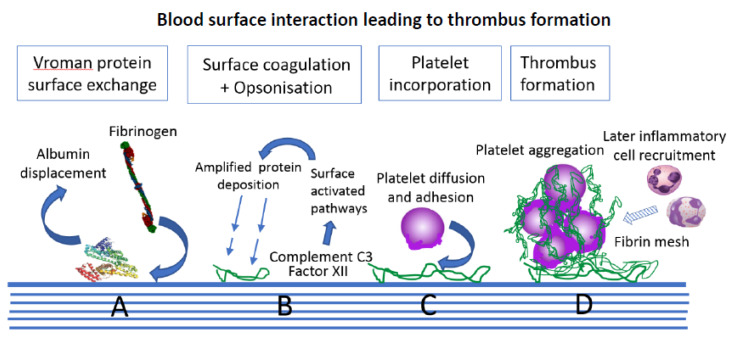
Schematic of surface coagulation sequence. (**A**) Initial rapid protein deposition, in milliseconds, subject complex, competitive displacement/remodeling via the Vroman effect, e.g., fibrinogen displacement of albumin. (**B**) Surface activated C3 and Factor XII trigger complement and coagulation cascades, leading to protein/C3b coating (opsonisation) and fibrin directed at the surface. (**C**) Platelets contact with coated protein sets of adhesion response. (**D**) Platelet adhesion leads to activation and promotion of fibrin clot, later inflammatory cells incorporated.

Following the protein interaction stage, the intrinsic coagulation pathway is initiated by Factor XII surface binding. Complement C3, the core driver of the separate complement cascade, causes independent protein coating and opsonisation. Complement C3 is triggered to fragment autocatalytically and produces C3b adsorbate for surface opsonisation; a surface that is now an attractant for inflammatory cells [45]. There is cooperation between the coagulation and complement pathways, and this later leads to the incorporation of inflammatory cells within the developing surface thrombus (Figure 4) [46,47].

The platelet is the specialist player of blood that really drives the development of a surface thrombus and it is later one of its most prominent constituents. Its study is difficult because of its environmental reactivity, including to the very surfaces used to handle it, and also its high sensitivity to shear stress. Moreover, its response to the developing surface coagulum is involves specific pathways triggered by specific surface receptor stimulation. The latter lead to dramatic morphological changes in the platelet, including degranulation, shape change to a discoid, and multiple bridging/aggregation. This super-structure of platelets and the entrapped fibrin then add to the growing thrombus [48]. For sensors, although the focus has been on surface chemistry, surface physical profile might also be important. In one proposal, surface roughness of platelet dimensions (~2 µm) was considered to offer a better match for platelet surface contact and, therefore, for thrombus formation than lower dimension roughness giving less platelet purchase [49]. Leukocytes in blood also become surface activated later [50], are then recruited into the thrombus, and promote further coagulation through cytokine release.

## 4. Tissue Oxygen Electrodes

### 4.1. Compartmental Difference

**Figure 5:**
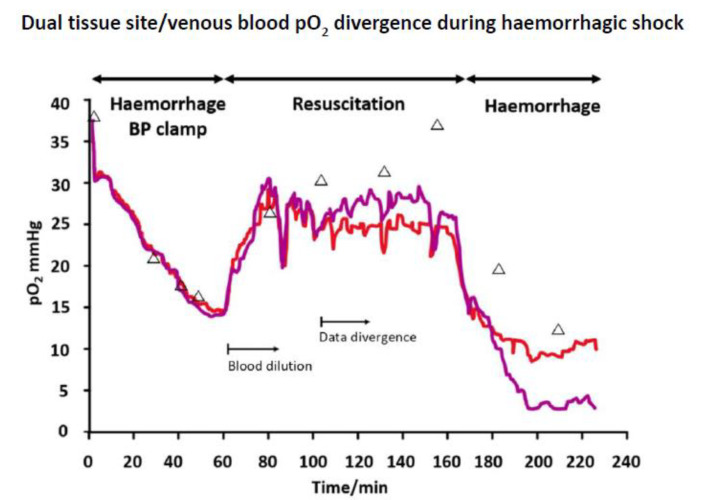
Tissue pO_2_ changes monitored in single rat during haemorrhagic shock. Sensors at matched implantation sites in flank. Initial haemorrhage clamped at reduced BP (40 mmHg); saline only resuscitation stabilises BP (60–70 mmHg); haemorrhage to exsanguination with extreme, terminal drop in BP. Resuscitation regimen would lead to cumulative blood dilution, progressively lowering oxygen carrying capacity to peripheral tissue. Adapted from [52].

The Clark pO_2_ sensor has also allowed for continuous monitoring of subcutaneous tissue pO_2_ [51]. Such a device has enabled the tracking of peripheral tissue pO_2_ during haemorrhagic shock [52], but there are indications that there are compartmental differences between blood and tissue. This is suggested for this study by an inter-sensor agreement that is greater than with venous blood (Figure 5). The measured tissue pO_2_ was significantly lower than that of blood at the later part of the shock experiment, a possible outcome of subcutaneous circulation shut down. Blood pO_2_ might, alternatively, reflect deeper tissue levels, e.g., of the more protected central organs, but further studies are warranted. Such changes cannot be readily decoupled from sensor drift, but if that was the cause, and then the polyurethane oxygen permeable membranes used would need to be exceptionally lacking in biocompatibility, and two hours had already been allowed for electrode stabilization.Post implantation stabilization periods are considered as artefact and certainly no clear explanation is given over their basis. However, the early drift seen during this run in period may well be a consequencet a tissue functional response, e.g., microvascular changes, to the intrusion. Mechanical tissue damage and microhaemorrhage will certainly also occur, but cannot be the full explanation. Calibration uncertainty in tissue lends uncertainty to true tissue pO_2_ values, which, in any case, will show local differences at the micron scale. Venous rather than arterial blood comparison was used for this study, though arterial pO_2_ is the benchmark for clinical use. Here, arterial changes were only observed at a very late stage haemorrhage; venous blood, derived from tissue, may more reflect tissue embedded sensor changes.

Gough reported the determinants of tissue pO_2_ under non-haemorrhagic conditions using a silicone membrane covered electrode [53,54]. Again, the similar stabilisation delay and uncertainties about tissue O_2_ were observed. They attributed variation in output at an array of tissue electrodes to local differences in vascular flow, and also observed slow to rapid fluctuations of tissue pO_2_, which they attributed to perfusion variation due to local vasomotor vascular control. The challenge with tissue is that of extracting valid physiological information in the face of an evolving tissue reaction, essentially a wound site. Surface biofouling raises the further uncertainty. A model for oxygen mass transport to the electrode was established [54], which indicated that local mass transport resistance limited the sensor response, whilst more remote oxygen delivery was rapid and associated with blood flows. The high permeability membrane used in this study allowed for the resolution of such extra-sensor effects. Over 13 weeks, these flux sensitive electrodes picked up tissue reactions that led to decay in local tissue O_2_ permeability, to ~10% of that in water. Even a collagen fibrous capsule build-up to 5 mm depth apparently did not entirely abolish diffusive transport. Whist such a high permeability experimental membrane can allow investigation of tissue effects, practical monitoring requires diffusion limiting membranes to negate external transport variables. Even here, however, over long time periods, it might prove difficult to achieve this if a substantive fibrous capsule forms. With tendon as a model dense collagen barrier, we found micro-solute diffusion to be just 1% of that in water [55].

### 4.2. Tissue Micro-Heterogeneity

**Figure 6:**
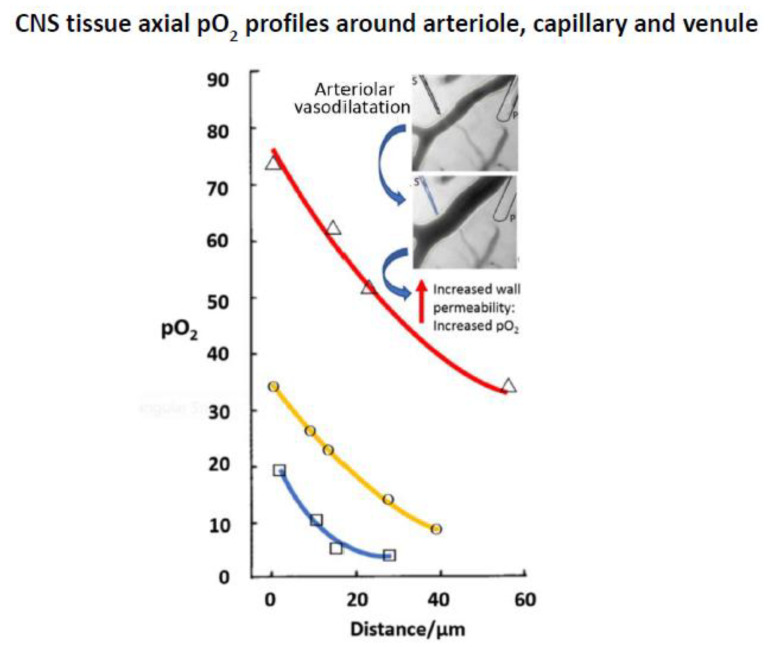
Rat cortical tissue pO_2_ at varying radial distances from (**-**) arteriole, (**-**) capillary, and (**-**) venule using 4 µm tip oxygen sensor showing exponential reduction with distance from vessel axis. Inset shows microsensor (S) on 30 µm diameter arteriole with subsequent vessel dilatation after delivery from nearby micropipette (P). Adapted from [58].

Tissue oxygen delivery distribution at a microscopic level is a field in its own right, and numerous mapping studies of pO_2_ have been undertaken using microelectrodes [56,57]. Oxygen micro-heterogeneity is variously a result of cell uptake, vascular delivery, and transport variation across the extracellular compartment. Cerebral tissue has been a particular focus for study because cortical blood vessels are more easily visualised, allowing for combined analysis of vascular organisation and pO_2_ distribution. In one study, a <5 µm diameter recess tip electrode with a collodion membrane was used to determine blood pO_2_ along a sequence of arteriole, capillary, and venule, together with perivascular tissue oxygen distribution [58]. This showed not only the expected pO_2_ reduction along the blood vessel cascade, but steep perivascular oxygen gradients extending ~60 µm into tissue giving pO_2_ reductions of up to 80% intravascular values (Figure 6). Muscular arterioles of the CNS are unique in providing through wall tissue oxygenation, so there were also gradients around these vessels. Such work offers insights that may be useful for neurosurgery giving a detailed picture of oxygen profile in CNS tissue. Additionally, the micro-delivery of vasodilator pharmacological agent to a single vessel was examined showing that with resulting relaxation of the arteriolar wall, through wall oxygen delivery was increased.

The question arises as to what macro-electrodes can tell us instead. They offer a ‘field of view’ extending hundreds of microns and, therefore, a sample aggregate of different tissue oxygen micro-sites, blurring the fractal complexities. The uncertainty is what the exact size of this sampling zone might be. The issue is typically bypassed by setting an empirical in vivo calibration against blood pO_2_ at the start. The outcome is still meaningful in that trend monitoring of pO_2_, is obtained for clinical purposes, egduring compromised tissue oxygen delivery [59,60,61] and in assessing cardiac oxygenation dynamics during ischaemia/reperfusion [62]. A commercial electrode is available for specialist CNS use (Licox, Integra Life Sciences Corporation) [63], but there is, as of yet, no general tissue clinical electrochemical sensor. This commercial system samples oxygen through an extended 18 mm^2^ area polyethylene tube so capturing changes across gross tissue regions. Again, uncertainties remain due to interrelationships between microcirculation organisation, flow, vascular distance, and mass transport, all balanced against cell metabolic uptake [64].

Beyond the validation of sensor stability using pre- and-post in vivo use calibration, there is no simple means of establishing the true pO_2_ experienced by a device [63]. This is where the need for disease correlates and diagnostic benefits diverge from the rigour of measurement science. An example of clinical value is in the case of head injury wheremeasured hypoxia appears to correlates with outcome. Some indication of the degree of uncertainty is shown by reported differences in monitoring output when the principle of measurements is changed, e.g., from electrochemical to optical, but these have been minor. 

The CNS is a relatively implant tolerant tissue, a contrast to subcutaneous tissue that demonstrates a florid, cellular inflammation. However, at the opposite extreme is when an inflamed tissue is deliberately monitored. The Licox probe was used to measure the pO_2_ of inflamed synovial tissue in rheumatoid and psoriatic arthritis patients [65]. Variable degrees of hypoxia were seen, and the degree appeared to correlate with disease severity. There was even the suggestion that hypoxia was a driver of inflammation, as manifested by the degree of oxidative damage coupled with the degree of hypoxia, and through the level of vascular damage and prevalence of T cells and macrophages in the inflammatory field.Consistent with this possibility was the improvement in oxygenation seen following anti-inflammatory therapy; pO_2_ doubled from ~20 mmHg in those who responded. A pO_2_ correlation was also demonstrated in relation to T cell, but not for B cell, infiltration, suggesting a disease causal link with the former. Any inflammatory milieu remotely like to this at a sensor implant site would radically change measured pO_2_, and no longer reflect systemic levels. Despite the oxygenation causal possibilty with the histology, there is also the likelihood that high respiring cells simply caused a low tissue oxygen and the measured levels were a reflection of the respiring cell population.

### 4.3. Cancer Tissue

Solid cancers grow rapidly and can outgrow their vascular supply, already compromised through disordered, dysfunctional blood vessels. Zones of hypoxia arise, which have a clinical relevance, because hypoxia confers tumour radio-resistance. A range of analytical techniques has been employed to study cancer blood supply and oxygenation and the field has been reviewed [66]. O_2_ microelectrodes, in particular, have made it possible to unravel the oxygenation architecture of cancer tissue. A commercial recess tip oxygen electrode (Eppendorf, Hamburg, Germany) housing a 17 µm gold cathode and a PTFE barrier layer enabled sequential tip tracking across tumour tissue at aligned 0.6 mm distances [67].

**Figure 7:**
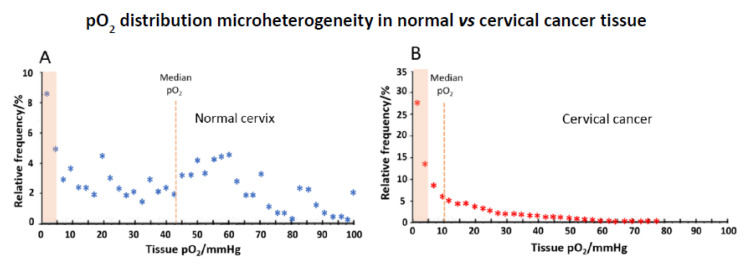
Percentage frequencies of pO_2_ measured across (**A**) Normal uterine cervix (seven patients, 432 samples), (**B**) Cervical cancer (150 patients, 13596 samples). Shaded areas highlight percentage prevalence of extreme tissue hypoxia of <5 mmHg. Each set of data represents combined data points from multiple patient samples. Adapted from [68].

This generated oxygen distribution data (histography), which when combined for tumours from many patients in the case of cervical tissue, showed a stark oxygen distribution difference for normal vs. cancer. Incervical cancer, for example, median pO_2_ was a mere 10mmHg, as compared with normal cervix at 43 mmHg, with also a huge preponderance (>60%) of exceptionally hypoxic microenvironments (Figure 7) [68]. It is notable that, even in normal tissue, sites of near zero oxygen levels do exist. Any hypoxia link to cancer prognosis appeared to be absent, but there is value, nevertheless, to such study of the oxygen state of cancers.

Tissue oxygen can also be monitored while using fibreoptic sensors. The principle of operation is fluorescent quenching as with the intravascular oxygen sensor. There is potential for application just as with electrochemical devices, and a widerange of critical care clinical applications have been considered [69]. When brain monitoring was undertaken, the optical affinity system gave slightly lower responses to an electrochemical device (Licox), and no advantage regarding response time was evident [70]. Differences that were seen variously related to device tip geometry, the area of the sensing surface, and how close it was juxtaposed to tissue, but evidently not to sensing principle. A possible discriminator of clinical relevance was the greater accuracy of the electrochemical probe at low pO_2_; this could be of value in monitoring brain hypoxia. Stabilisation times is long, and this would affect operational deployment of either device in an emergency situation. Yet again, transduction chemistry is far less important than design.

## 5. Glucose Electrodes

### 5.1. Monitoring Needs

Glucose remains the most important target for continuous monitoring. Whilst detailed electrochemical studies have reported on the mechanisms and optimisation of enzyme-electrode electron exchange, for example, these alone cannot alone translate into operational benefit. Commercial development has helped to advance the latter operational aspect, initiated previously through single use strip technology [71]. Diabetes has now escalated in importance since the early days of glucose sensor development, and so glucose sensing has risen up the health priority list, along with this, the interest in continuous monitoring. Diabetes now poses a massive healthcare burden currently affecting 9% of the global adult population. Within this group are the 5-10% Type I diabetics [72] needing insulin who warrant closer monitoring to manage their therapy. They are also liable to marked glucose fluctuations, e.g., during concurrent illness when insulin sensitivity changes. The brittle diabetes sub-population lies within this group, and though small, has highly unpredictable insulin needs and difficult to manage glucose levels; these patients are prone to dangerous hypo-/hyperglycaemia [73]. Accordingly, continuous glucose offers specific benefits to the Type I diabetic, with a reduction of long-term vascular and other complications through improved glucose control.

Sensors when used as single use devices allow for greater design flexibility permitting, for example, genetically modified enzyme use, leachable/soluble mediator, and a host of modifie working electrode surfaces. The major consideration here is mass usage, shelf life stability and calibration-free measurement. Beyond that, survival in blood need only be for a few seconds. An in vivo sensor, by contrast, can be allowed high calibration variabilty pre-use, but, beyond that, stability during use has to be sustained over long periods, and the electrode surface, enzyme component and any incorporated reagent components have to be guaranteed to be safe, or unable to be released. Hence, permitted flexibility over design chemistry is limited. Additionally, intravascular sensors risk microthromus dissemination, so, despite its uncertainty, tissue is the near-universal target for continuous glucose monitoring (CGM).

### 5.2. Clinical Realities

Measurement based on O_2_/H_2_O_2_ transduction of the glucose oxidase (GOD) reaction forms the basis of all CGMs:

(3) $$\mathrm{Glucose}+O_{2}\to \mathrm{Gluconolactone}+H_{2}O_{2}$$

By avoiding additional leachable chemical components and mediators, thisreagentless approach complies withobligatory requirements for invasive use. Local toxicity, teratogenic, and other adverse effects have also to be avoided based both on the general precautionary principle, and, regulatory compliance. Unfortunately, the glucose K_m_ for glucose oxidase does not allow for simple application to clinical glucose levels without barrier membranes exerting control over glucose/oxygen access to the enzyme layer. Given the dual substrate kinetics of the enzyme, that such membranes have been developed with any success is an understated achievement. The oxidase not only depends vitally on adequate, freely available, oxygen co-substrate, but its ambient levels then also dictate the apparent glucose K_m_. In the discourses on kinetic pO_2_ effects at the enzyme, it is often forgotten how precariously limited this oxygen is in terms of actual concentration. At a normal arterial pO_2_ range of 80–100 mmHg, to a first approximation, Henry’s Law indicates oxygen concentrations of a mere 88–102 µM. This is distinctlyreaction limiting, below V_max_ conditions of oxygen despite the low oxygen K_m_ of 180–950 µM (BRENDA) for the enzyme. Apparent glucose K_m_ is significantly lowered by O_2_ limitation under in vivo conditions. Moreover, this can be on a background of a possible down shift in K_m_ due to a diffusion limitation in the enzyme layer. Barrier membranes are firstly required to diminish glucose access to the enzyme so sensor output is at the lower end of the Michaelis–Menten saturation curve and, therefore, linear. Thereby, the enzyme also becomes less of an oxygen sink with better maintenance of microenvironmental oxygen concentrations. Notwithstanding this, O_2_ transport also has to be differentially advantaged, otherwise a reduced response with an unextended glucose K_m_ results. The membrane design challenge is to achieve this in the face of low transmembrane oxygen gradients. Any benefit here through faster intrinsic oxygen diffusivity is limited; diffusion coefficients in water are glucose 6.7 × 10^−6^ cm^2^/s, oxygen 20 × 10^−6^ cm^2^/s. Membranes with mosaic, composite, or porous strictures are frequently used, accordingly. We are yet to achieve predictive membrane design here despite the bofy of work on membrane modeling and innumerable reports on enzyme kinetics.

As a transduction principle, the second generation glucose biosensor offers a distinct advantage; it removes the key variable of oxygen control. Its signature characteristic is its integral redox mediator, which, when optimised for low potential, mostly also avoids extraneous electrochemical interference. The first generation device, operating at a typical +0.65 V vs. Ag/AgCl, also necessitates a molecular weight discriminating inner membrane as a barrier to any species larger than H_2_O_2_. This, however, offers added physical protection from electrode poisoning by diffusible biochemical molecules, especially those with thiol moieties. No such protection is possible with the second generation device, and this may matter for long term monitoring. Moreover, leachable or soluble mediator is precluded for in vivo application.

The exception to the in vivo sensor reagentless paradigm has been the osmium electron shuttle that was developed by Heller [74], now used for clinical CGM (FreeStyle Libre, Abbott, Almada, CA, USA). This uses mobile pendent pyridine groups along linear polymer chains for stable retention of osmium (III/II) redox centres, whilst allowing their dynamic interaction to create a relay to the working electrode. The sensor is accepted for 14 days monitoring use, and ultra-low (~2%) drift independent of calibration has been reported [75], considered to be on the basis of a high biocompatibility covering membrane. Even during normal dynamic glucose changes, there was stated to be concordance with blood glucose values. However, this system, enters a new type of unknown into the measurement; devices are already manufacturer calibrated to generate automatic blood equivalence [76]. This brackets a series of known variables, including blood vs. tissue glucose relations within and between patients, glucose dynamic change modulation of this relationship, and also any implant site dependence. Studies have shown that all are variables affecting response, and they should be considered as factors that need to be allowed for on an individual basis. Nevertheless, the clinical value of this approach has been recognised through improved monitoring benefit to the patient.

Generally, in the literature the true measured glucose value in tissue is bypassed, as with oxygen, and the starting point for data recording is after calibrated against blood in vivo. This is really a form of data ratioing across compartments rather than a true calibration, and it provides no actual information regarding the tissue state. Modeling of plasma-tissue exchanges by contrast recognises delayed and variable exchange kinetics and the need to factor in genuine lag times to underpin data correction [77,78].

Added to the physiological uncertainties, there are changes due to the reactivity of tissue at the implant site. As of yet, no material has been able to claim the stealth performance needed to eliminate the disruptive tissue reaction, despite the many sensor design iterations [79,80,81]. Performance decay is also maximum in the hours following implantation; the so-called run-in period of hours to days, which warrants separate consideration.

### 5.3. Membranes and Coatings

The coatings and coverings for glucose sensors have mostly used existing materials. The key requirement is low surface fouling and stable glucose and oxygen permeability. Shichiri et al. [82] were the first to demonstrate such packaging in their use of polyurethane in an implanted device. We and others have similarly utilised polyurethane [83,84]. Such repurposing of a medical polymer helps to reduce the unknown risk of a new material and with appropriate porosity and diffusion control enables a response that is sufficiently independent of sample physical properties or oxygen background for the clinical glucose range. However, commercial CGM manufacturers have been able to develop and incorporate new materials, as reviewed by McGarraugh [85]. The Guardian Minimed (Medtronic) employs a block copolymer polyurethane with a glucose permeable hydrophilic diol phase for glucose, balanced against a silicone that would presumably be O_2_ only permeable; the DexCom (DexCom Inc., San Diego, CA, USA) uses a hydrophobic/hydrophilic polyurethane mixture for balancing diffusive transport with a presumably similar differential permeability intended. The minimisation of any oxygen diffusion limitation for the enzyme reaction is part of the design goal for materials here. In the absence of a mediator membrane transport selectivity provides an important means of achieving this. The design challenge is the micromolar levels of oxygen concentration in tissue, likely to be below arterial values. The FreeStyle (Abbott) departs from the polyurethane platform and uses a functionalised vinyl pyridine-styrene copolymer, but the mediator based device here is, in any case, O_2_ independent. One interesting claim made for the latter was of the unprecedented absence of any tissue encapsulation, even after one year implantation, [80], though the muscle location here might have been a factor.

Membrane innovations have also been reported for CGMs on an experimental basis. Moussey has advanced a range of compositions that have variously included a hydroxypropyl methacrylate hydrogel coating on polyurethane that reduced inflammation and fibrosis [86], humic acid films that provoked less tissue reaction [87], and a structurally robust epoxy-polyurethane, which, though leading to a fibrous capsule, also stimulated vascularisation [88]. In one study, a porous polyvinyl alcohol scaffold was used as a covering matrix over the sensor, and this took up collagen growth from the tissue surround, along with inward growth of new blood vessels. However, the collagen barrier effect countered the blood supply benefit of increased vascularisation [89]. Nafion, a perfluorosulphonic ionomer, has been extensively studied and, as a tissue contacting sensor surface, it has generated a reduced tissue reaction with only a thin fibrous capsule at three months [90]. A comparative study of negatively charged membranes as part of a sol gel layer, espectively, utilised Nafion, dextran sulphate, and polystyrene sulphonate [91]. The results were similar for these, with thin collagen capsules being seen at 12 weeks foreach material. The lack of a difference is a reminder that chemical refinement does not necessarily change the outcome. Neutral polyethylene glycol (PEG) has well recognised antifouling properties and, as a tissue contact surface, provoked less tissue reactivity with a reduced local cellularity and tissue adherence [92].

Phosphoryl choline as an outer cell membrane zwitterion has been used to reduce protein and cell deposition in blood at an intravascular glucose sensor located in the carotid artery of rats [93]. This was an acute study, and long-term outcome would need to be investigated.

Application has been transferred to tissue. Following initial combinatorial screening, a PEG crosslinked phosphoryl choline polymer was used over a commercial CGM sensor in mice and primate studies [94]. Inflammation mitigation by the phosphoryl choline reduced the need for repetitive in vivo calibration. Blood to tissue glucose mismatch was reduced, although there was still late fibrous capsule development. Phosphoryl choline translation from blood to tissue would be a valuable future direction. Here and for other studies, the possibility cannot be excluded of changed surface mechanics, especially with a gel. Tissue is reactive to surface mechanical cues. Whilst not necessarily due to mechanical change, in one study, soft electrospun gelatin coatings on polyurethane fibres over sensors reduced fibrous encapsulation, as compared with non-coated fibre [95].

**Figure 8:**
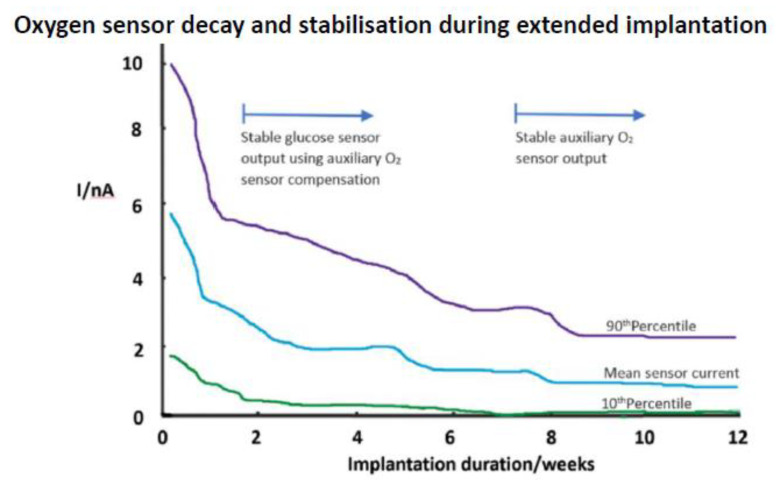
Auxiliary oxygen sensor current decay in subcutaneously implanted glucose sensors in pigs. Data represent one week moving averages of daily mean sampled currents and the spread of data for 60 electrodes. Adapted from [96].

As an alternative to the registration of H_2_O_2_ product, Gough has advanced the use of cathodic O_2_ measurement. In one study, a surgically fully implanted sensor was operated for a year [96]. A dual sensor arrangement was necessitated here, with a second, non-enzymic, O_2_ sensor compensating for background tissue oxygen variation. Glucose oxidase was used in a crosslinked gel and, whilst there was a tissue reaction and a steep response decline with the secondary oxygen sensor (Figure 8), the dual O_2_ approach enabled glucose monitoring after two weeks. The wide statistical spread seen in responses for different oxygen sensor implants indicates the variability of the local tissue response. Whilst such a protracted stabilisation delay is an option, it would only seem so if long term implantation is contemplated, and that demands a high level of confidence in a sensor that needs surgical implantation. A subsequent six month human study with this sensor demonstrated stable oxygen compensated glucose tracking [97]. The collagen capsule imposed response delay was of the order of 10 min, so workable for clinical purposes.

Boronic acid is capable of reversible binding with saccharides and a diboronate system with an attached fluorophore sensitive to glucose binding induced conformational change has been reported for intravascular glucose monitoring [98]. This is an attractive possibility because of the theoretical independence from the need for ongoing glucose mass transport during measurement. However, there was a need for gel containment of the affinity molecule with membrane barriers to prevent access to micro- and macrosolutes into the affinity phase. A special need was for an outer Pt modified membrane to degrade damaging low level peroxides from blood. Response times of over 5 min. would not be particularly slower than most electrochemical sensors. No interference was seen with potential blood constituents, and use of membranes eliminated the effect of blood haematocrit on response. Correlation data, whilst acceptable, appeared no superior to thatseen for tissue electrochemical sensors; whether the affinity principle provides for greater reliability and independence from fouling would need detailed comparator studies. However, it is unlikely that an electrochemical glucose sensor would ever be a practical proposition for intravascular use; the intended use of the optical sensor was critical care monitoring. Injectable boronic acid gels with non-invasive optical tracking have been reported for glucose, but these constitute a rather different type of monitoring strategy, subject to the challenges of safety and biocompatibility as well as reliable signal extraction through tissue.

### 5.4. Bioactive Molecule Release for Biocompatibility

Ahyeon, et al. reviewed drug loaded membranes [99]. Dexamethasone, a high potency steroid, can suppress inflammation and late fibrosis; VEGF (vascular endothelial growth factor) can promote vascularisation to augment glucose delivery. However, biological complexities may becme evident. Vallejo-Heligon et al. [100] found dexamethasone loaded polyurethane to both suppress inflammation and to promote neovascularisation, extending the sensor operating period; however, its combination with VEGF led to the depression of VEGF stimulated neovascularisation [101]. Despite a high early implantation effectiveness of such loaded membranes, eventual reservoir depletion is a potential drawback, especially given the use of thin, low capacity membranes. One study instead delivered VEGF from a cannula while using an osmotic pump [102]. Neovascularisation was demonstrated, evident at least 40 days at a tissue distance of 1.3 cm. Composite material designs might also extend function life time. Dexamethasone when loaded onto poly (lactic-co-glycolic acid) (PLGA) microspheres of two molecular weights provided early and late release with bioactivity retained for six months [103]; this wasconditioned by the differential degradation rates of the two polymers. With microspheres embedded within porous polyvinyl alcohol (PVA), any surface fouling was offset by porosity recovery as the microspheres degraded [104].

Bioactive NO releasing membranes have also been developed [105] with the aim of suppressing pro-inflammatory cytokine release and thereby inflammatory cell recruitment. Stabilised sensor operation depended again on a maintained NO reserve. Interestingly, here, the sensor run-in period was short. A NO pharmacological effect might offer a clue to some altered tissue response, with perhaps a link to cell signalling. NO is electroactive at anodic voltages, but, if its interference is constant, then monitoring would be possible. In regard to this, the authors postulated that the variable generation of endogenous NO by inflammatory tissue might contribute to signal instability.

The widening repertoire of clinical therapeutic agents, including biologics, should provide a new generation of anti-inflammatory agents. Masitinib, a small molecule agent used to treat mast cell tumours, is one example. Mast cells are central to the tissue response; they are immediately deployed in inflammation, undergo ready degranulation, and through this variously release proinflammatory cytokines, serotonin, and histamine, and thus accelerate inflammation. Masitinib works by blocking mast cell receptors, thereby suppressing intracellular kinase signalling and stabilising the cell membrane. However, when used as a released agent in a sensor [106], the protective effect was limited, certainlynot as much as might have been expected from drug potency.

### 5.5. Tissue Reactivity to Implants

For bulk dependent implant devices, the tissue reaction is of a lower order concern, but for surface response dependent sensors, even a minor reaction can have profound effects. When observed in the opposite sense, tissue is actually the more sophisticated sensor. Thus, it has mobile surveillance through its constituent cells, a strong capacity for recognising, even the smallest of foreign body intrusions as ‘non-self’ and an ability to resolve shape; whatever way any three-dimensional (3D) object is packaged, or disguised it is readily recognised. From this recognition starting point, a cascade is established, embodied in the Foreign Body Reaction. This is designed to degrade the intrusion, and failing that, to package and isolate it behind a fibrous capsular wall. It is too fundamental a part of the armoury of an organism, linked to its very survival, to be readily countered. While the outcome for the technology is seriously adverse, for the biology it is an unmitigated success. The current state of the art is that, whilst we have dissected the complex response pathways, it is our understanding of these that lags behind [107,108].

**Figure 9:**
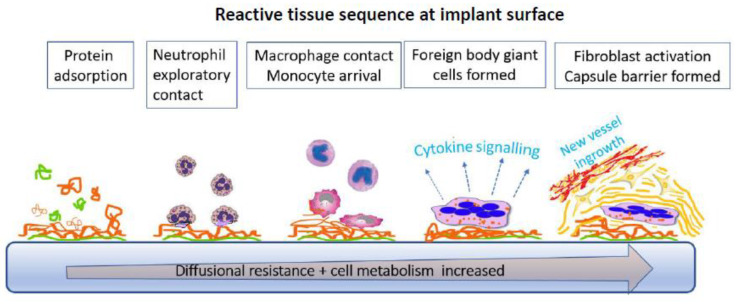
Schematic of tissue foreign body response in sequence: (**1**) Rapid protein deposition masks sensor surface; deposited layer increases. (**2**) Tissue neutrophils sense the surface and send chemotactic signals, mast cells promote inflammatory background. (**3**) Macrophages accumulate with population reinforcement by blood monocytes. (**4**) Failure to degrade surface stimulates more powerful multinucleated giant cell formation from macrophages, with enhanced signalling. (**5**) End stage of more quiescent collagen formation and cumulative barrier formation by fibroblasts with parallel neovasularisation.

On implantation in tissue, as in blood, rapid protein deposition takes place and the device is already packaged by a layer that, though conditioned by the original surface, itself then goes on to condition the subsequent response (Figure 9). Early protein reorganisation, layer accumulation, and denaturation characterise this initial growing protein layer. The tissue cellular tissue response is subsequently affected by specific receptor binding to the adsorbed proteins. The first responders are exploratory polymorphonuclear leucocytes (neutrophils) and, with mast cells, they initiate diffuse chemotactic signalling to attract other phagocytic cells (macrophages). These amplify the directional signalling and recruit even more macrophages. The bioactive factors released include PDGF (platelet derived growth factor), TNF-α (tumor necrosis factor alpha), and IL-6 (interleukin-6). To this mix are added monocytes from blood, together with proinflammatory mediators, replenishing the macrophage pool. If phagocytosis against the device fails, macrophages on the surface fuse to make more effective multinucleated foreign body giant cells, through a trigger that is unknown [109]. There is also an outpouring of degradative diffusible agents with no other purpose than to solubilise the intrusion, which includes acidic cell contents, oxygen free radicals, and hydrolytic enzymes. Ultimately, degradation might be a highly desirable biomaterials outcome if the agent is a surgical suture, but, if it is a sensor membrane, it becomes a clear problem. Preferential degradation of the soft segments of a polyurethane used for glucose sensors illustrates this [110].

If the implant stimulus persists over days or weeks, a more cellularly heterogenous inflammatory cell architecture is built up with added lymphocytes and plasma cells. Healing then ensues if there is no outright toxicity. This progresses behind a cell layer adjacent to the device, and variously hosts a dense network of inwardly directed blood vessels, fibroblasts, and macrophages; this is granulation tissue. This is also the remodeling phase of the response, and a precursor to final collagen capsule deposition by fibroblasts. This sequence of events around a non-toxic implant is pre-programmed constant, and refractory to control with only its quantitative aspects varying across different materials [111] or through suppression regimens.

### 5.6. Tissue Reaction Implications for Glucose Sensors

Some microhaemorrhage is inevitable during any implantation. The locally released RBCs can then become a sink for glucose, though not for oxygen. After early RBC removal and the entry of more actively metabolising nucleated cells, glucose and oxygen access to the sensor can both become reduced. The direct injection of macrophages to a sensor implant site reproduced this effect [112], but, interestingly, but not lymphocyte injection despite the equivalent metabolic activity of these cells. Within days, a rapid population change occurs with an order of magnitude expansion of neutrophil number followed by decay and a commensurate increase in lymphocyte number. No differences in this tissue response sequence was seen in one study, regardless of whether or not the sensor was operational and releasing toxic H_2_O_2_ into tissue [113]. What is a constant with all devices is that final fibrous capsule formation is inevitable and it becomes the arbiter of what is then ‘seen’ by the sensor. Studies of beyond a week confirm such capsular development and its effect on glucose exchanges, in one example leading to a 24 min. response lag following intravenous glucose [114]. The result was also consistent with the arrival time of injected fluorescent glucose analogue. True physiological glucose exchange between blood and tissue is considered to be quite rapid, requiring < 5 min. for completion, so implanted sensors clearly create an artefactual delay.

The collagen capsule, far from being a simple, static accumulation of collagen fibres, is an evolving structure with its own vascular network and an internal palisade of cells apposed to the sensor. Novak et al. [115] took this structural duality into account in their modeling of glucose transport. They concluded that lag time was determined by capsular thickness, whilst sensitivity was a function of capsular porosity and local vascularity. Additional effects of macrophages and adipocytes metabolism were small here. By contrast, glucose losses due to local cell metabolism were evident in a cell loaded fibrin gel, and further accentuated by exposure to a pro-inflammatory agent [116], again highlighting cellular influence on an inflammatory matrix.

**Figure 10:**
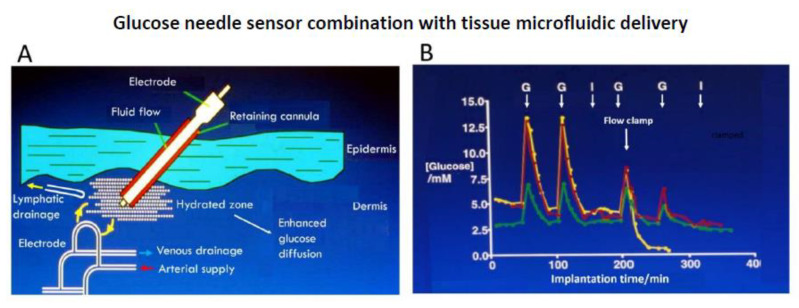
(**A**) Schematic of subcutaneously implanted glucose needle electrode within open ended cannula for delivering fluid around the implanted sensor to create a limited hydrated zone. (**B**) Subcutaneous glucose monitoring in rat (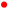) venous blood glucose, tissue glucose at 60 µL/h microflow (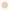) and at a constrained flow of 10 µL/h (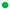) showing underestimated glucose and total loss of response with clamped flow. Bolus tail vein administration of glucose (G) and insulin (I). Adapted from [83].

Our own studies on implant sensor stability have led us to a materials independent strategy. Whilst inflammation comprises a hypercellular environment with distinct histological characteristics, it is also a zone of high, histologically silent, fluid influx due to permeabilised capillaries delivering protein rich fluid, passaged then to the lymphatics, and also part returned to the microcirculation. A balance of hydrostatic and osmotic transcapillary pressures drives the fluid flow, as embodied in the Starling mechanism [117]. Our approach here was simply to deliver extra protein-free fluid to the implant site. This utilised an electrode-cannula coaxial arrangement for low volume fluid delivery around the sensor tip (Figure 10A). Subcutaneous tissue has a negative hydrostatic pressure, and the arrangement used pumpless tissue driven flow. The resulting locally reduced protein load both reduced sensor fouling and stabilised response. The response lag time with respect to blood was eliminated and unusually, the measured tissue levels matched blood without in vivo correction.

Accordingly, far from local glucose being diluted, glucose access was likely to have been enhanced through the well hydrated, open structure, interstitial tissue space (Figure 10B) [83]. Hence, the conclusion of the approach is that a fluidized zone with rapid glucose exchanges with the blood compartment is obtained. As both a practical and model system, fluid management of the interstitium could provide an alternate means of manipulating the implant inflammatory environment.

## 6. Lactate

As the end stage metabolite of anaerobic respiration, lactate offers a quantitative measure of hypoxic and shock states where the peripheral tissue O_2_ supply is compromised. As such, it has formed a core means of tracking the severity of such states and their response to treatment. It is inevitably subject to rapid change, but, despite this, clinical continuous monitoring is not available. Some experimental work on lactate oxidase sensor based in vivo monitoring in brain has been conducted. Here, oxygen co-substrate limitation at the enzyme could potentially lead to underestimated lactate levels, so an oxygen discriminating membrane has been one option [118]. Alternatively, stoichiometric regeneration of oxygen from H_2_O_2_ product has been tried using incorporated CeO_2_ catalytic particles, and have improved lactate response during brain monitoring in hypoxic rats [119]. As with glucose, without an independent reference measurement in tissue, true tissue lactate level is difficult confirm. Direct validation is possible in the case of an intravascular sensor, and anti-thrombotic NO release from such a sensor for added device stabilisation has been reported with rapid response to both lactate and hypoxia [120]. However, with subcutaneous deployment, a substantially blunted and delayed response was seen. Moreover, in this pig model, upper vs. lower body implantation altered responses. We have also found a blood-tissue discrepancy in experimental shock with subcutaneous electode site dependent output [121].

The scale of the tissue mismatch well exceeds that seen with glucose and suggests that, at least under shock conditions, there might be an added barrier to lactate release from the circulation. This should not occur at the capillaries, which are not selective for micro-solute, but possibly in the interstitial tissue space, which, as a polyelectrolyte, might create an ionomeric barrier to the lactate anion. In diffusion through cartilage, we found the diffusion coefficient for ascorbate anion to be a small percentage of that for similar size neutral molecules [55]. The complexities of blood tissue compartmentalisation were shown in a study of muscle interstitial tissue [122]. Here, microdialysis sampling demonstrated tissue lactate at rest to be double that of plasma water at rest, butlevels converged with plasma during exercise elevations, whilst glucose at rest was about half that of plasma, but again converged with plasma during exercise. The results indicated that muscle can control its extracellular environment. Subcutaneous connective tissue will not have this capability. Our exercise study with subcutaneous tissue microdialysis did not make a similar comparison, but it showed a blunted lactate response, even delayed to the post-exercise period during which tissue glucose appeared to falling [123]. Therefore, it is clear that study of different interstitial locations and comparisons between techniques for lactate are needed to help understand intercompartmental exchange, for if we do not understand these, our understanding of events even for the traditional blood compartment will be limited.

## 7. Conclusions

*Practical* in vivo sensors are a unique sub-set of electrochemical sensors, and they constitute a distinct practical offering when compared to the fundamental electrochemistry studies on, say, cell signaling molecules and CNS neurotransmitters. Unlike many sensor types, including industrial, they operate not merely in a hostile environment, but one that is active, reactive, and protean in its nature. This rather counters the idea of biology as a benignmatrix presenting mild solution conditions. The recruitment of high surface activity and destructive cells in high numbers renders the implant site far from representing the normal physiological state locally and provides evidence of a contrived effort at sensor disruption. However, our quest for data immediacy on some variables in the acutely ill patient requires just such sensors.

There also remains a need for repertoire expansion into a broader palette of intermediary metabolites, as these interact dynamically, and they will give added clinical information. Currently, we better understand that, because of compartmental differences, there is an even stronger case for developing the tools for examining these separate entities at different locations.

Electrochemical sensors have been the mainstay of such endeavours, and this review has highlighted the insights that they have given us. This reinforces the need to resolve the generic problem of biocompatibility. It has been an inappropriate quest in many ways to search for the single material or surface that absolves us from this problem—the result has simply been more model systems. The quest needs to be far more deeply rooted in the study of the reactive biology. If nothing else, we have learned that this reactivity is not surface restricted and has a signalling hinterland remote from the surface.

By addressing the right issues, electrochemical sensors will be able to expand their service from physiology to precision medicine. Additionally, future development of closed loop feedback control and autonomous therapeutic management will become feasible. Much of this capability is in place, including sensing chemistry, but it is the biological control of the implant site that remains to be resolved. A far better, multi-parameter, understanding of the individual’s dynamic bio-signature might emerge from this, and would be in step with the needs of individualised therapy, currently advancing through genomic profiling.

The implications for future development are that, from our experience of in vivo operation, it is now clear that there are generic material and membrane needs that have to be satisfied to provide the correct contact surfaces in vivo and through this a way of reducing the body’s reactivity. This might be only partially achieved, as has been evident with biomaterials more generally, but still tailoring of the device packaging now should be the priority. Success here can help to bring the field forward, as has been evident in the case of implantable electronics. Semi-implantable sensors should be focused on as these can allow ready removal and replacement. Further work with miniaturisation and multiple arrays through microfabrication should also be undertaken by way of creating more reproducible systems and for evolving less intrusive sensors. This will provide for greater measurement confidence through multiple redundancy. What seems less of a need is the invention of ever more chemistries; transduction advances will not deliver practical value without the interfacing and miniaturisation effort. The review has only focused on a few target species, but, from the point of view of critical care, they are entirely sufficient. If, finally, we can achieve a paradigm shift by creating reversible label-free bioaffinity then a whole span of protein, hormone, metabolite and drug species come into scope. The lack of rapidly reversible immunobinding may be a problem for in vitro *assay*, but it is a major drawback to dynamic monitoring. It is, perhaps, time to also look around for rapid reversibility systems that exploit cell membrane receptor principles.
